# Electrode Cooling Effect on Out-Of-Phase Electrothermal Streaming in Rotating Electric Fields

**DOI:** 10.3390/mi8110327

**Published:** 2017-11-06

**Authors:** Weiyu Liu, Yukun Ren, Ye Tao, Xiaoming Chen, Qisheng Wu

**Affiliations:** 1 School of Electronics and Control Engineering, Chang’an University, Middle-Section of Nan’er Huan Road, Xi’an 710064, China; liuweiyu@chd.edu.cn (W.L.); qshwu@chd.edu.cn (Q.W.); 2 School of Mechatronics Engineering, Harbin Institute of Technology, West Da-zhi Street 92, Harbin 150001, China; xiaomingchen@stu.hit.edu.cn; 3 State Key Laboratory of Robotics and System, Harbin Institute of Technology, West Da-zhi Street 92, Harbin 150001, China

**Keywords:** electrothermal flow, rotating electric field, out-of-phase smeared structural polarization, electrode cooling, external natural convection, co-field flow rotation

## Abstract

In this work, we focus on investigating electrothermal flow in rotating electric fields (ROT-ETF), with primary attention paid to the horizontal traveling-wave electrothermal (TWET) vortex induced at the center of the electric field. The frequency-dependent flow profiles in the microdevice are analyzed using different heat transfer models. Accordingly, we address in particular the importance of electrode cooling in ROT-ETF as metal electrodes of high thermal conductivity, while substrate material of low heat dissipation capability is employed to develop such microfluidic chips. Under this circumstance, cooling of electrode array due to external natural convection on millimeter-scale electrode pads for external wire connection occurs and makes the internal temperature maxima shift from the electrode plane to a bit of distance right above the cross-shaped interelectrode gaps, giving rise to reversal of flow rotation from a typical repulsion-type to attraction-type induction vortex, which is in good accordance with our experimental observations of co-field TWET streaming at frequencies in the order of reciprocal charge relaxation time of the bulk fluid. These results point out a way to make a correct interpretation of out-of-phase electrothermal streaming behavior, which holds great potential for handing high-conductivity analytes in modern microfluidic systems.

## 1. Introduction

The rapid advance of lab-on-chip technology requires the exploitation of new approaches capable of achieving precise fluid actuation and manipulation at the micrometer scale [1,2,3,4]. Incorporation of microelectrode structure into confined microfluidic channels stands for an opportunity to exert ponderomotive electrokinetic force either on the suspension medium itself or colloidal particles monodispersed within [5,6,7,8]. Research of electrohydrodynamics (EHD) has captured more attention from the microfluidic community than ever before [9,10,11,12,13,14,15].

In continuous-phase microfluidics, there exist mainly two categories of EHD flow: electroosmosis (EO) [16,17] and electrothermal (ET) [18,19]. As for EO, both linear and nonlinear electroosmosis are originated by diffuse charge dynamics adjacent to an electrical double layer (EDL) of nanometer thickness at a solid/electrolyte interface [20,21]. The specific difference between the two electroosmotic techniques comes from the origin of surface charge density on the sharp material interface: direct current (DC) electroosmosis is due to native surface free charge chemically adsorbed on insulating channel sidewalls [22]. However, induced-charge electrokinetics is related to the polarized bound charge at the outer surface of Stern layer induced around polarizable dielectric materials in a background electric field [23,24]. The action of a tangential field component on the free charge within the diffuse screening cloud gives rise to electroosmotic slip flow that is full-developed right outside the Debye layer, and nonlinear electroosmosis can even survive in a low-frequency alternating current (AC) electric field due to the field-induced nature of its EDL charge [25]. For electroosmotic devices of both subtypes, however, since the active driven forces merely exist within EDL of nanometer distances from the charged boundary and not throughout the entire volume of micrometer-thick fluid layer, undesired backflow is very likely to take place at slightly larger distances from the charged channel sidewalls [26].

Unlike EO that moves directly on charged surfaces, induction EHD stems from smeared structural polarizations of inhomogeneous liquid medium [27]. Specifically, gradients of solution electric properties are usually induced by non-uniform heating of fluid medium due to either internal Joule heating [28,29] or external heat source from artificial thermal elements [30,31,32,33]. Interaction of such heating-induced dielectric gradients with an applied electric field effectively produces a kind of dielectrophoretic (DEP) force exerting directly on the fluid bulk. In light of this, induction EHD is commonly referred to as AC electrothermal flow (ACET) [34]. There are manly two practical prototypes for actively driving ACET fluid motion, including standing-wave electrothermal (SWET) and traveling-wave electrothermal (TWET) [35]. SWET fluid motion is due to in-phase bulk polarization in a stand-wave electric field without change in phases, which can be produced over microelectrode arrays with neighboring electrodes oppositely polarized, and SWET often behaves as multiple vortex flow pairs on electrode surfaces [36,37]. On another hand, TWET streaming is due to out-of-phase bulk polarization in a traveling-wave (TW) electric field with spatial phase variations, which can be generated over interdigitated multi-phase metal-strip electrode array with continuous phase transition along the consecutively distributed microelectrodes, and TWET usually behaves as horizontal streaming flow parallel to the signal-phase propagation [38]. Since there is as least one electrode intrusion between oppositely polarized electrodes in a typical four-phase induction device, SWET and TWET cannot coexist in those traditional configurations with linearized electrode arrays, which intrinsically restrains the rich diversity of ACET flow obtainable in microfluidic systems.

**Figure 1:**
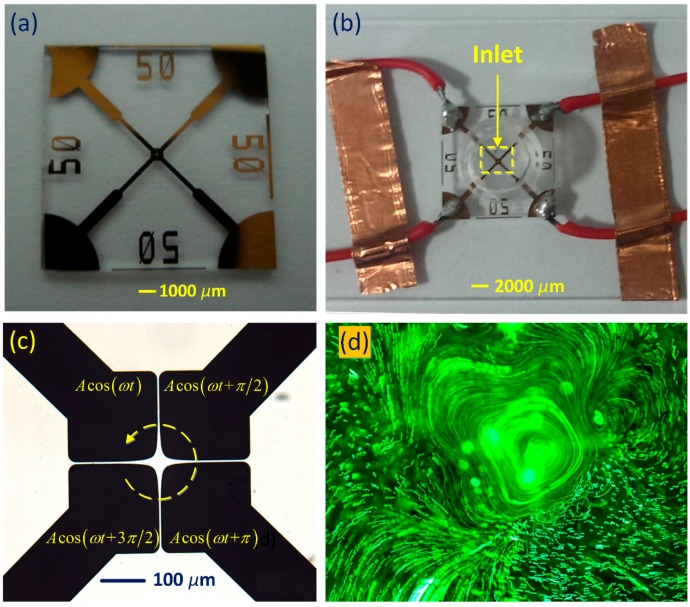
Experimental setup of the microfluidic chip for studying the behavior of electrothermal flow in a rotating electric field (ROT-ETF). (**a**) An optical micrograph of the four-phase polynomial electrode configuration employed in this work; (**b**) A picture of the experimental microfluidic device where Plant Design Management System (PDMS) microchamber is plasmally bonded to the glass substrate with desired circulating electrode patterns; (**c**) Schematic of the applied 90°-phase-shifted sinusoidal voltage waves that produce a counterclockwise rotating electric field above the cross-shaped interelectrode gaps; (**d**) The fully-developed co-field TWET induction whirlpool above the electrode array at the Debye frequency of electrolyte suspension.

To handle this issue, we developed a device geometry with four-phase polynomial electrode array configured in a circulating style in this work (Figure 1). TW field over such a circulating array is not traveling in a linear route but rotates along a circular trajectory in synchronous with the field frequency, so we call this form of wave propagation as a rotating electric field because of its circularly polarized feature [39,40]. Different from the conventional linear TW array, electrodes of opposite phases are positioned right against one another with quite small space intervals in this circulating electrode design, so both SWET and TWET appear and actively compete against one another within a broad frequency range, which brings additional richness to existing ACET techniques [41]. Consequently, it would be of particular interest and great benefit to study the behavior of electrothermal flow in rotating electric fields (ROT-ETF) where both in-phase and out-of-phase polarizations occur and vary as a function of applied field frequency. First and foremost, we experimentally observed the performance of driving ROT-ETF in our device and discovered that the central out-of-phase TWET whirlpool above the cross-shaped interelectrode gaps rotates in the direction of signal-phase propagation, which violates the actuation of anti-field induction EHD on a glass base predicted by previous researchers [42]. Specifically, multiple SWET vortex flow pairs are induced in perpendicular orientation to the electrode plane within low frequency ranges, while merely one dominating horizontal co-field TWET whirlpool is captured above the glass base at field frequencies on the order of the reciprocal Debye relaxation time of the bulk fluid. In addition, the rotating flow profile becomes more circular and less helically cascading with further increase in field frequency. Subsequently, according to preliminary mathematical analysis, we developed different heat transfer models to present a reasonable explanation of this attraction-type induction vortex. Surprisingly, this peculiar flow reversal phenomenon is originated by external natural convection on millimeter-scale highly-conductive electrode pads that substantially gives rise to an electrode cooling effect, which simultaneously makes the rotating flow velocity measured by experiments much closer to theoretical prediction compared to the traditional non-cooling model. These unique characterizations of our electrothermal microdevice, including both the rotation and pump motions of microfluidics under the effect of electrode cooling, ingeniously provide new insights into tackling major issues that involve on-chip operations at low temperatures, such as conducting chemical reaction [43], drug delivery [44] and cell culture [45] in the context of high-conductivity buffer solutions for modern micro total analytical systems.

## 2. Materials and Methods

### 2.1. Basic Design of the Circulating Electrode Structure

Complicated multilayer fabrication procedure has to be implemented for manufacturing a metal-strip TW electrode array [46]. To evade complex interconnections, we make use of a coplanar polynomial-shaped electrode design to produce a traveling potential wave circulating above the channel bottom surface (Figure 1). By applying 90°-phase-shifted sinusoidal voltage signals to the typical four-electrode configuration with increasing phase in the clockwise direction, a TW electric field is produced above the gaps and propagates anticlockwise in the direction of decreasing field phase. The transient potential imposed on the sequential electrodes is *V*_1_ = Acos(ωt), *V*_2_ = Acos(ωt+90°), *V*_3_ = Acos(ωt+180°), and *V*_4_ = Acos(ωt+270°), respectively. Here, *A* is the voltage amplitude, *ω* is the angular field frequency of the applied voltage wave (Table 1). On this basis, electric field vector at the center of circulating electrode array is approximately of the following form:

(1) E=E0Re((ex−jey)ejωt)=E0cos(ωt)ex+E0sin(ωt)ey

Such an electric field of constant magnitude $E_{0}$ ≈ 2*A*/*d* revolves with a circular trajectory in the direction of the signal-phase propagation, and is referred to as a “rotating electric field”. Here, *d* = 50 μm denotes the nearest distance between opposing electrodes of 180° difference in voltage phase.

### 2.2. Chip Fabrication

We fabricate the 4-quadrant electrode array for studying ROT-ETF by standard microelectronic technique. After finishing the design of photo mask, EPG533 photoresist layer (Everlight Chemical Industrial Corporation, Taiwan) is spin-coated on a glass slide, which then experiences Ultraviolet (UV) light exposure, development and post-baking in sequence. Subsequently, a 10 nm-thick Cr layer and 100 nm-thick Au layer are magnetically sputtered onto the photoresist pattern one by one. A lift-off process is carried out to obtain the final pattern of metal electrode layer on the glass substrate. At the same time, a Plant Design Management System (PDMS) microchamber is fabricated using standard soft lithography technique, and assembled onto the glass base with polynomial electrode patterns by plasma bonding, eventually giving rise to the microfluidic chip employed in current work, as shown in Figure 1.

### 2.3. Sample Preparation and Experimental Setup

In the experiment, we employ KCl electrolyte solution as the working fluid, as made by adding KCl electrolyte into Deionized (DI) water medium. A conductivity meter is applied to monitor the solution ionic strength until the electric conductivity increases up to σ = 0.05 S/m. The electrolyte bulk has a characteristic charge relaxation frequency $f_{c1}=\sigma /2\pi \varepsilon$ = 11 MHz, beyond which displacement current begins to dominate over Ohmic conduction. 500 nm-diameter fluorescent latex spheres are suspended in the electrolyte solution at a moderate number concentration to keep track of the electrothermal fluid motion. A commercial multi-phase function generator (TGA12104, TTi, Buckinghamshire, UK) was employed to produce sinusoidal voltage waves in a broad frequency range from 1 MHz to 35 MHz. Four-phase AC voltage signals are applied to the four circulating electrodes so that there is a 90° phase shift between the AC signals imposed on every adjacent phase. Waveform of these phase-shifted voltages is monitored by a multi-channel digital oscilloscope (TDS2024, Tektronix, Beaverton, OR, USA).

After injecting the fluorescence bead suspension into the microchamber and switching on the anticlockwise rotating electric field, rotating motion of tracer particles is observed in real time by an optical microscope (BX53, Olympus, Tokyo, Japan). During the experimental observation, focuser knob of the optical microscope should be adjusted at first, to make the electrode array most clearly presented. At this time, it is assumed that the focus plane is right on the surface of coplanar electrode array. Since the flow rotation due to ROT-ETF occurs at a certain height away from the electrode plane, the focuser knob need another regulation to make the observation plane levitated a vertical distance of 70 μm (on the order of electrode separation) above the electrode array on the basis of previous adjustment.

**Figure 2:**
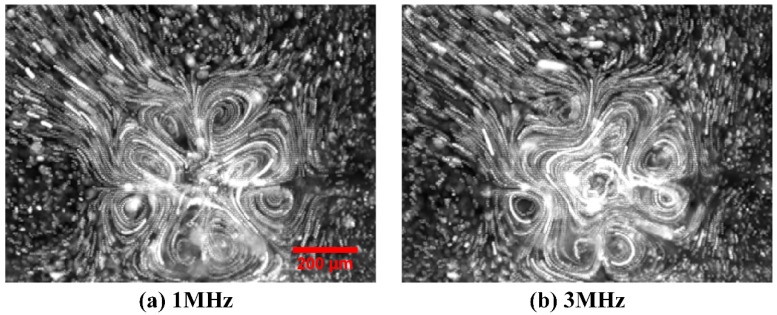

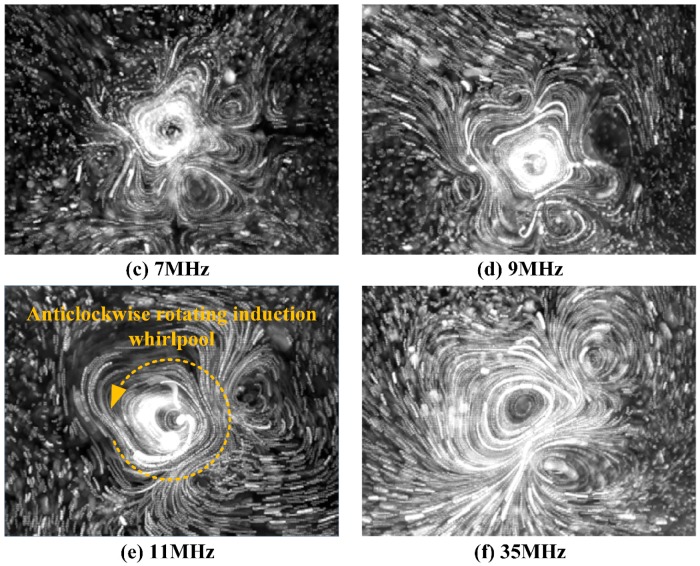
Frequency-dependent flow field of ROT-ETF captured in experiment using a high-speed CCD camera, for given voltage amplitude *A* = 7.5 V. (**a**) *f* = 1 MHz; (**b**) *f* = 3 MHz; (**c**) *f* = 7 MHz; (**d**) *f* = 9 MHz; (**e**) *f* = 11 MHz (relaxation frequency of the dielectric dispersion); (**f**) *f* = 35 MHz. With increasing field frequency beyond the reciprocal charge relaxation time, rotating streamlines of the anticlockwise induction whirlpool become more circular and less helically cascading downward.

We take consecutive snapshots with a high-speed charge-coupled device (CCD) camera (RETIGA2000R, Qimaging, Surrey, Canada) and then superimpose the image frames in a software called ImageJ. The experimental images with particle streamlines due to superimposition operation are shown in Figure 1d and Figure 2. Horizontal rotating speed of latex beads due to the action of out-of-phase TWET streaming is calculated manually by dividing the physical distance moved for a particle by the time elapsed under the assistance of ImageJ software, with the measurement results shown in Figure 6a,b. The standard deviation for each data point was obtained by five repeated measurements under the same experimental conditions.

### 2.4. Flow Components of Electrothermal Streaming in Rotating Electric Fields

In a circularly-polarized rotating electric field, sufficiently large electrolyte conductivity can induce a vertical bulk temperature gradient through Joule medium heating. This results in inhomogeneous electric properties of liquid medium across the thin fluid layer. Such dielectric gradients interact with the applied rotating field, inducing a volumetric free charge distribution that lags behind the propagation of applied voltage wave. These charged ions experience a Coulomb force in the same electric field which forces them into electrophoretic motion, and drag the surrounding fluid along through viscous effect. The bulk polarization process described above effectively leads to electrothermal vortex flow on the phase-shifted polynomial microelectrode array. According to the basic theory of Maxwell-Wagner structural polarization, ROT-ETF includes two sets of EHD flow components [41]:

(1) TWET component due to out-of-phase induced polarization

Out-of-phase component of the induced polarization generates one horizontal induction vortex rotating along the *z* axis above the circulating electrode array. The TWET whirlpool is maximized if the period of AC voltage waves is commensurate with the characteristic relaxation time of the dielectric dispersion process [47,48,49]. That is, the horizontal flow rotation induced by the vertical electrorotational torque is most evident at Debye frequency of the fluid medium $f_{c1}=\frac{\sigma }{2\pi \varepsilon }$ for onset of bulk ionic screening.

(2) SWET component due to in-phase induced polarization

Since electrodes with 180° phase difference are placed opposite to one another in the four-phase polynomial electrode configuration, potent SWET flow component behaving as multiple vortex pairs in perpendicular orientation to the electrode plane can be produced by the in-phase component of applied voltage wave. The vertical SWET streaming possesses a low-frequency conductivity plateau where motion of free charge dominates, as well as a high-frequency permittivity plateau where dielectric polarization plays an important role. Since the Coulomb force and dielectric force counterbalance one other, SWET vortex pairs across the thickness of fluid layer vanish at a characteristic crossover frequency $f_{c2}=\frac{\sigma }{2\pi \varepsilon }\sqrt{\frac{-\beta }{\alpha }}=\frac{\sqrt{5}\sigma }{2\pi \varepsilon }$ [41]. Because flow field of SWET in DC limit is much stronger than that at high field frequencies, the horizontal TWET rotating whirlpool appears to dominate over the vertical SWET vortex pairs at *f* > *f*_c2_ = $\frac{\sqrt{5}\sigma }{2\pi \varepsilon }$. In addition, within low-frequency ranges *f* < *f*_c1_, fluid motion of TWET whirlpool diminishes, while that of vertical SWET streaming enhances, which would exert a negative impact on the rotating flow pattern of out-of-phase induction vortex. Specifically, the horizontal TWET whirlpool shrinks in size, and exhibits more helical flow streamlines cascading downward at lower field frequencies.

### 2.5. Computational Model

Two different heat transfer models are developed herein to reconstruct the heat transfer process across the gold microelectrode arrays for dealing with distinct experimental conditions:

(1) None-cooling condition

Since thickness of the electrode layer is extremely thin (~100 nm) compared to other geometric dimensions in the experimental chip, the electrodes are treated as transparent media for heat transfer. That is, both temperature and normal heat flux are continuous across the electrolyte/substrate interface, as if the circulating electrode array does not exist at all.

(2) Cooling condition

The electrodes are made of gold material in our device, which is of thermal conductivity k_Au_ = 340 W·m^−1^·k^−1^, considerably higher than that of water solution k_water_ = 0.6 W·m^−1^·k^−1^ and glass substrate k_glass_ = 1 W·m^−1^·k^−1^, so that the electrode structures can be treated as ideal thermal conductors which are effectively isothermal bodies. Then, for inputting voltage signals from function generator to the microelectrode arrays, external wire connection has to be achieved by fabricating large-scale electrode pads of millimeter dimension, as shown in Figure 1a,b. In similar device configurations, heat exchange between metal electrode bars and ambient environment has a propensity to occur due to external natural convection. Since gold electrodes are good thermal conductors, they can transfer cooling energy from ambient environment to the device internal, which would make the electrode bars not only an isothermal body but also fixed at the referential temperature T_0_ = 293.15 K of atmospheric condition for a sufficiently large heat transfer coefficient. That is, because gold microelectrodes of excellent heat dissipation capability are connected to external wires by large-scale metal pads, electrode cooling due to external natural convection ought to be taken into consideration and modeled by setting the electrode surface at the ambient temperature T_electrode_ = T_0_ = 293.15 K in the simulation analysis.

A commercial FEM software, Comsol Multiphysics 5.2 (COMSOL, Stockholm, Sweden), is used to solve the mathematical boundary-value problem for theoretically obtaining the electrothermal flow field, and detailed governing equations and boundary conditions have been presented in our previous work [34]. For the circulating electrode array, shape of the polynomial electrode edge is represented by the following hyperbolic equation:

(2) $$\left\{ \begin{array}{l} x=d/2\sec\theta \\ y=d/2\tan\theta \end{array}\right.\;-\pi /2<\theta <\pi /2$$

**Figure 3:**
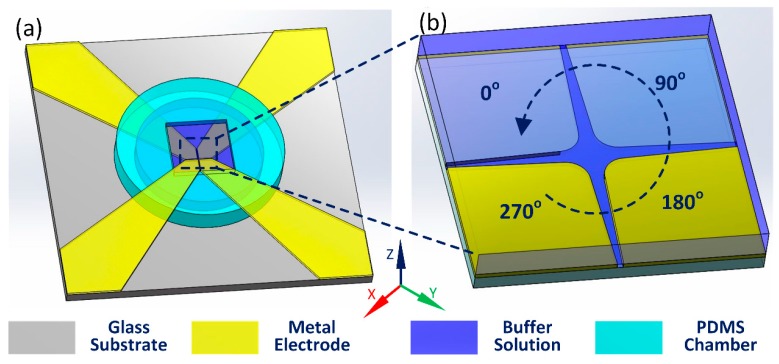
Construction of the 3-D simulation model: (**a**) Schematic of the 3-D computational domain; (**b**) A top view of the four-phase polynomial electrode configuration.

Rotating the hyperbolic curve by 45° around the *z* axis, we obtain the electrode pattern in the first quadrant. Subsequently, by conducting several steps of mirror imaging operations, the electrode pattern within the entire *x*-*y* plane is digitally established, as shown in Figure 3b. The non-isothermal harmonic electric field, heat transfer and electrothermal flow field are solved in a fully-coupled manner using the enhanced ACET model derived in [34,50]. In the numerical simulation, we only need to calculate the electric field and fluid motion in the electrolyte solution, while the energy balance equation for temperature field has to be solved within the entire fluidic device, including the fluid bulk, glass substrate and patterned gold electrodes (Figure 3a):

(a) Glass substrate of 500 μm in thickness

This domain has a thermal conductivity k_glass_ = 1 [W·m^−1^·k^−1^], with the bottom surface of the fluidic device set at ambient temperature due to strong natural convection at the microscope platform.

(b) 500 μm-thick liquid layer

The electrolyte solution has an electric conductivity 0.05 [S/m], dielectric permittivity ε = 7.08 × 10^−10^ [F/m] and thermal conductivity k_water_ = 0.6 [W·m^−1^·k^−1^], where several large-voltage effects including nonlinear Joule heating source, temperature-dependent dynamic viscosity and improved electrothermal body force are taken into account [34,50].

(c) Four-quadrant polynomial electrodes

The electrode array is applied with 90°-phase-shifted TW voltage signals in sequence, resulting in a counterclockwise-propagating rotating electric field. Accordingly, the complex amplitude of AC voltages imposed on the four electrodes corresponds to *A*, *jA*, -*A*, -*jA*, respectively, with *j* denoting the imaginary unit.

## 3. Results and Discussion

### 3.1. Experimental Observation of ROT-ETF Fluid Motion

First and foremost, electrothermal flow profile in a broad frequency range from 1 MHz to 35 MHz is experimentally observed for a given voltage amplitude *A* = 7.5 V (Figure 2 and Figure 6a). Then, the voltage-dependent flow velocity of the horizontal TWET induction vortex is measured and quantified for field frequency fixed at the reciprocal Debye relaxation time of medium bulk, *f* = $f_{c1}=\frac{\sigma }{2\pi \varepsilon }$ = 11 MHz (Figure 6b). As shown in Figure 2, at field frequency exceeding 11 MHz, the horizontal induction vortex rotating along the *z* axis is large in size and has quite circular streamlines at the center of rotating field (Figure 2f). On the contrary, at field frequency lower than the inverse charge relaxation time of fluid bulk, size of the central whirlpool decreases (Figure 2a–d), due to, in part, a strengthened action of vertical SWET streaming. The TWET whirlpool rotates anticlockwise all the time, in the direction of field propagation (Figure 2e), and can be regarded as a typical attraction-type induction vortex. On this basis, the electrode plane should have a lower temperature than the medium bulk. Due to the high thermal conductivity of Si material, a silicon substrate will make the Joule heating-induced hot spot levitate some distance above the electrode surface, which naturally leads to the formation of an attraction-type device (Figure 3a in [34]). In current experiment, glass material of low thermal conductivity k_glass_ = 1 [W·m^−1^·K^−1^] is used to construct the insulating base for supporting the circulating electrode array. The low heat dissipation capability of glass material effectively makes the hot spots due to electric heating generation located right on the electrode surface, namely, the maximum temperature rise takes place right in the electrode plane, thereby actuating a repulsion-type induction vortex (Figure 3b in [34]). In this sense, preliminary theoretical analysis goes against the counterclockwise rotating whirlpool captured in our experiment with a high-speed CCD camera.

### 3.2. Electrode Cooling Effect on Rotating Direction of Induction Whirlpool

Perch-Nielsen et al. [42] predicted that flow direction of induction pump using a glass substrate is against the signal-phase propagation. The low thermal conductivity of glass material cannot effectively remove the internal electric heat generation to the ambient environment, so that the hot spots of the whole microfluidic system are positioned within the interelectrode gaps right on the channel bottom surface. According to the canonical classification of induction EHD, repulsion-type out-of-phase streaming occurs when electrolyte solution at the electrode surface has higher electric conductivities than other regions within the medium bulk, which is in qualitative agreement with the previous simulation results on TWET pumping from Perch-Nielsen et al. [42].

In this work, however, experimental observation indicates that the TWET whirlpool rotating around the central *z* axis is in the direction of signal-phase propagation. That is, both the rotating electric field and horizontal electrothermal vortex rotate along the counterclockwise direction, which is equivalent to an attraction-type induction pump. For this reason, the channel bottom surface on the glass substrate ought to have a lower temperature than the fluid bulk on top of the electrode array in this microfluidic device, in order for current fluidic chip to be in good analogy with attraction-type devices (Figure 3a in [34]). It has been forecasted that induction pumping is in phase with the sinusoidal voltage wave under the condition with a silicon base, since the high thermal conductivity of silicon material k_si_ = 140 [W·m^−1^·k^−1^] is able to remove the internal Joule heating to exterior of the device with high efficiency. Under such circumstance, the maximum temperature rise takes place in the medium bulk at some vertical distance above the electrode array rather than right on the channel bottom surface. Accordingly, electrolyte solution on the electrode surface has a lower conductivity than that at the hot spots on top of the electrode array, resulting in an attraction-type induction fluidic device.

Although glass substrate is employed in this study, we observed attraction-type out-of-phase electrothermal streaming, which is identical to the situation using a silicon base. In the simulation analysis of Perch-Nielsen et al., Fourier heat conduction on the electrode array was ignored, taking into account a negligibly small thickness (100–200 nm) of the thin electrode layer usually involved in lab-on-chip [42]. In typical microsystems, highly conductive materials, such as gold, platinum and so on, are routinely used for depositing microelectrode patterns on insulating substrates, those noble metals are not only conducting for electric current but possess high thermal conductivities as well, e.g., that of gold attains as high as k_Au_ = 340 [W·m^−1^·k^−1^].

For inputting voltage signals from function generator to the microelectrode arrays, external wire connection has to be achieved by fabricating large-scale electrode pads of millimeter dimension, as shown in Figure 1a,b. In similar device configurations, heat exchange between metal electrode bars and ambient environment has a propensity to occur due to external natural convection. Since gold electrodes are good thermal conductors, they can transfer cooling energy from ambient environment to the device internal, which would make the electrode bars not only an isothermal body but also fixed at the referential temperature of atmospheric condition for a sufficiently large heat transfer coefficient. Due to the cooling effect brought by external natural convection, the electrode surface becomes the plane of low temperature, and region of higher temperature elevation is raised up with a bit of vertical distance from the channel bottom surface, resulting in an attraction-type induction device where the rotation of TWET whirlpool is in the direction of signal-phase propagation.

**Figure 4:**
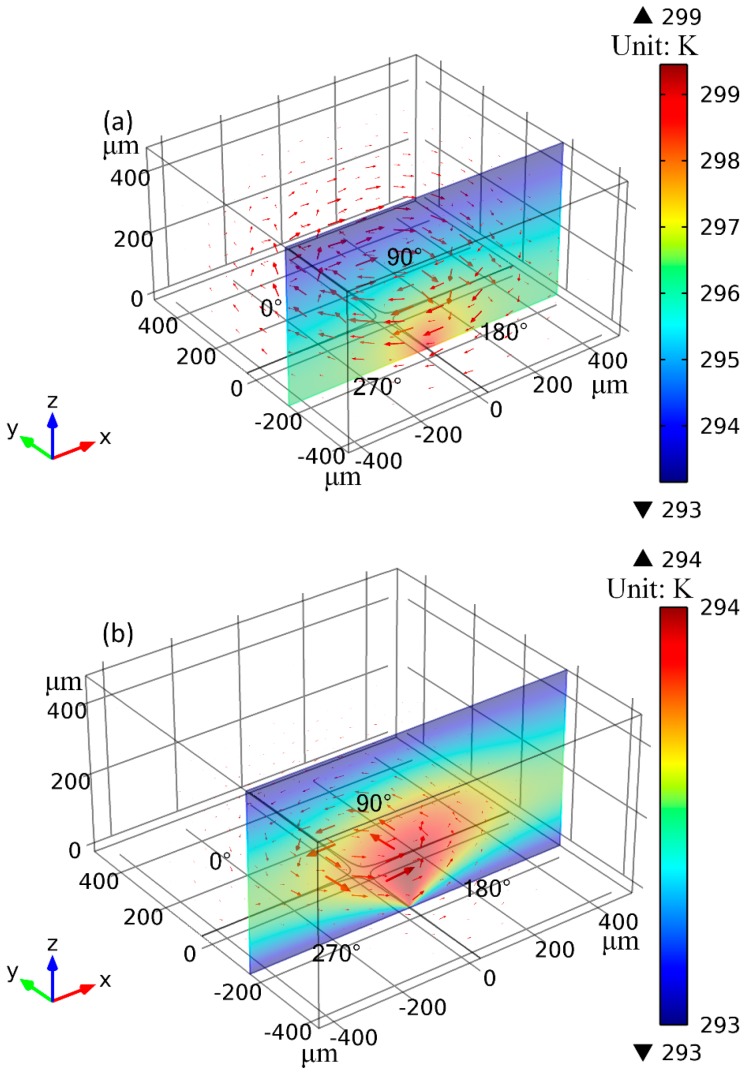
Influence of external natural convection-induced cooling of the gold microelectrodes on temperature field distribution and rotating direction of the central TWET whirlpool (unit: K). (**a**) A volumetric arrow plot of electrothermal flow field and *x*-*z* cross-sectional surface plot of internal temperature field at *y* = −200 μm by ignoring the exchange heat flux on the electrode pads, where the anti-field induction vortex rotates clockwise; (**b**) Under the influence of evident electrode cooling, out-of-phase induction EHD streaming transits from repulsion-type to attraction-type which is in the counterclockwise direction of signal-phase propagation.

Figure 4 illustrates the simulation results of temperature field as well as the ACET flow field, by calculations using different thermal conjunction conditions (including the non-cooling and electrode cooling conditions indicated in Section 2.5) at the electrode surface. Gonzales et al. [41] derived self-similar analytical solutions of rotating electrothermal flow field in an ideally vertical temperature gradient caused by external illumination. The temperature field due to internal Joule heating is of a radial pattern (Figure 4), however, so it is reasonable to analyze Joule-heating-induced ROT-ETF by direct numerical simulation.

As shown in Figure 4a, by ignoring heat conduction through the electrode array, the temperature maxima are located right at the cross-shaped interelectrode gaps on the channel bottom surface, resulting in clockwise rotating TWET vortex opposite to signal-phase propagation, i.e., an equivalent repulsion-type induction device. As shown in Figure 4b, after incorporating the cooling heat flux of gold microelectrodes by external natural convection, the internal hot spots due to Joule heating are levitated a bit of vertical distance away from the electrode plane, resulting in anticlockwise-rotating TWET vortex, which is in the direction of rotating electric field and can be well compared to an attraction-type induction device. As a result, the co-field rotating induction whirlpool from calculation is in qualitative agreement with the anticlockwise electrothermal streaming captured by experimental observations. Accordingly, though the thickness of gold microelectrode array (~100 nm) is much smaller than other geometry dimensions (10–500 μm), its cooling effect on the entire microfluidic device cannot be safely dropped, which usually gets overlooked by most of previous researchers.

### 3.3. Comparison between Theory and Experiment

**Figure 5:**
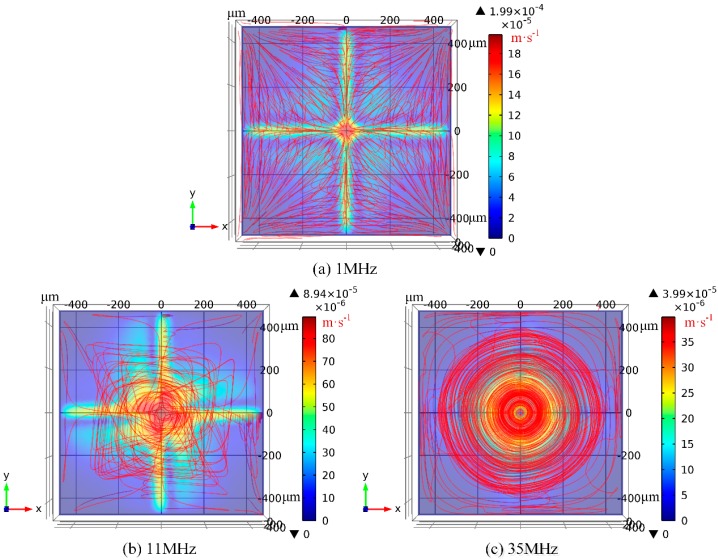
Simulation results of ROT-ETF flow field under distinct field frequencies where electrode cooling effect is taken into account: (**a**) *f* = 1 MHz for low-frequency limit; (**b**) *f* = 11 MHz at the characteristic dispersion frequency; (**c**) *f* = 35 MHz within the high-frequency range.

In the presence of electrode cooling, electrothermal flow profiles at different field frequencies are calculated by conducting numerical simulation, as shown in Figure 5. There are eight vortex flows in perpendicular orientation to the electrode plane at *f* = 1 MHz (Figure 5a), with flow patterns in good accordance with experimental observation in Figure 2a. Since *f* = 1 MHz is much lower than the medium charge relaxation frequency *f*_c1_ = 11 MHz, the multiple small in-phase SWET micro-vortices rotating orthogonal to the electrode surface are induced by the oppositely polarized electrodes with 180° phase difference and dominate over the horizontal out-of-phase TWET vortex. In Figure 5b, one large induction whirlpool is generated at the relaxation frequency, and it is not possible to find the vertical SWET flow components any longer. The out-of-phase induction vortex cascades downward with a helix flow profile, which coincides well with our experimental measurement in Figure 2e. According to simulation result in Figure 5c, the electrothermal flow field is completely stipulated by the horizontal out-of-phase TWET whirlpool at *f* = 35 MHz, with quite circular rotating streamlines. Since the field frequency 35 MHz surpasses the characteristic crossover frequency $f_{c2}=\sqrt{5}\sigma /2\pi \varepsilon$ = 24.6 MHz for SWET, the SWET flow component in perpendicular orientation to the electrode plane decays considerably compared to the low-frequency limit, that is, the horizontal co-field TWET whirlpool governs the electrothermal flow field within the entire fluidic chamber at such high field frequencies. Consequently, the rotating electrothermal streamlines seem no longer to spiral downward, which can be validated by the experimental observation in Figure 2f as well.

**Figure 6:**
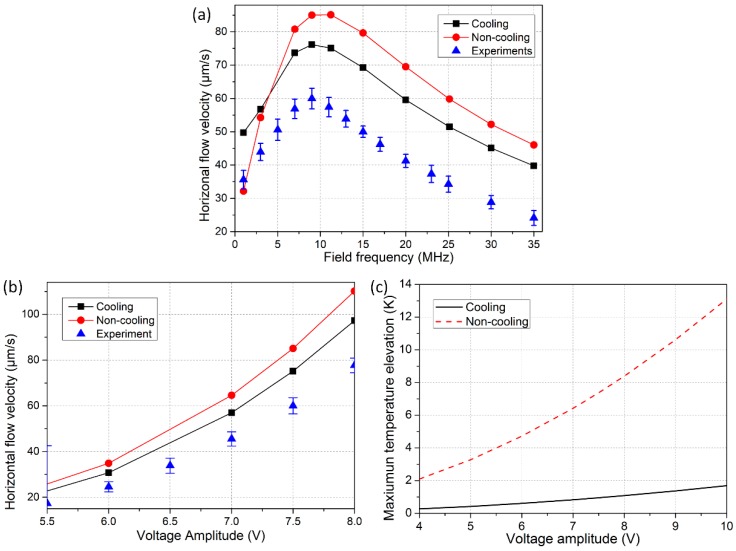
Comparison of electrothermal flow velocity and temperature elevation between theory and experiment for both cooling and non-cooling cases. (**a**) Frequency-dependence of maximum horizontal flow velocity; (**b**) Voltage-dependence of maximum horizontal flow velocity; (**c**) Voltage-dependence of maximum temperature rise within the medium bulk.

Theoretical prediction and experimental measurement of the maximum horizontal flow velocity at height *z* = 70 μm are compared for the situations with and without electrode cooling in Figure 6a,b. The frequency-dependence in Figure 6a indicates that external natural convection imposes no significant impacts on the dispersion characteristic of out-of-phase smeared structural polarization. That is, the induction vortex in a rotating field exhibits a single Maxwell-Wagner structural dispersion at charge relaxation frequency of the bulk medium. In correspondence to the analytical expression of induction pump force (Equation (16) in [34]), magnitude of rotating flow velocity in ROT-ETF is a Lorentz function of the applied field frequency $u\propto \omega \tau /\left(1+{\left(\omega \tau \right)}^{2}\right)$, so the fastest TWET whirlpool takes place at the reciprocal Debye time ω=1/τ = 11 MHz for onset of bulk ionic screening. With field frequency deviating from the dispersion characteristic, the rotating fluid motion decreases in a very rapid manner, as shown in Figure 6a.

The maximum horizontal flow velocity from theory is 85 μm/s without electrode cooling, and it is lowered down to 75 μm/s in the presence of conspicuous external natural convection. Both of them, however, are larger than the characteristic flow velocity 60 μm/s captured in our experiments. The reason behind may include two aspects: on one hand, because of the high electrolyte conductivity involved, strong electrochemical reactions can occur at the electrode/electrolyte interface even beyond the reciprocal resister-capacitor (RC) time constant for induced double-layer charging, which may suppress the healthy development of electrothermal induced flow. On another hand, the actual situation of internal heat transfer is definitely more complicated than current considerations. Anyway, the improved heat transfer model, which incorporates the electrode cooling effect, can well account for the co-field rotating electrothermal vortex in phase with the signal-phase propagation, and flow velocity predicted from the cooling model is closer to experimental measurement than that given by the non-cooling model. For this reason, in the context of applying an appropriate heat transfer model, the improved ACET theory is able to capture the basic feature of ROT-ETF.

As indicated by Figure 6b, the voltage-dependence of TWET rotation velocity is clearly a nonlinear function. The fluid motion enhances dramatically for large applied voltages and even exhibits a fourth-power growth trend. In fact, under the condition of electric heat generation acting as the main heat source (implying $\Delta T\propto \sigma E^{2}$), we can see that the induced volumetric charges including both free and bound are proportional to the third-power of field intensity ($\rho \propto \Delta T\cdot E\propto \sigma E^{3}$), and thereby the flow velocity scale increases with fourth-power of the applied voltages $u\propto f_{ET}\propto \rho E\propto \sigma E^{4}$. In this sense, ACET is a nonlinear electrokinetic phenomenon, and survives well in the high-frequency circularly-polarized electric field used in current work. Different from the quartic voltage-dependence growth trend for internal heating, the flow velocity would have a quadratic voltage-dependence under the condition that external heat source (artificial heat element or illumination) dominates [41]. In Figure 6c, the maximum temperature elevation without electrode cooling is much higher than that with appreciable external natural convection, which fits well with the actual situation. For this reason, quicker out-of-phase rotating whirlpool is induced in the non-cooling condition compared to that with electrode cooling effect (Figure 6b), due to a higher temperature elevation in the bulk fluid (Figure 6c).

### 3.4. Numerical Prediction on the Effect of Electrical Conductivity

As has been analyzed previously, for reconstructing the correct flow direction of the co-field induction whirlpool in rotating electric fields, we can approximately deem that the isothermal body of electrode array has a temperature value T_0_ = 293.15 K of the ambient environment, due to, in part, external natural convection on the large-scale electrode pads of millimeter dimension for external wire connection. Under such electrode-cooling effect, the temperature field as well as the electrothermal flow field were calculated for different electrical conductivities of the suspending medium, where Fourier heat conduction in the presence of Joule medium heating has been taken into account.

**Figure 7:**
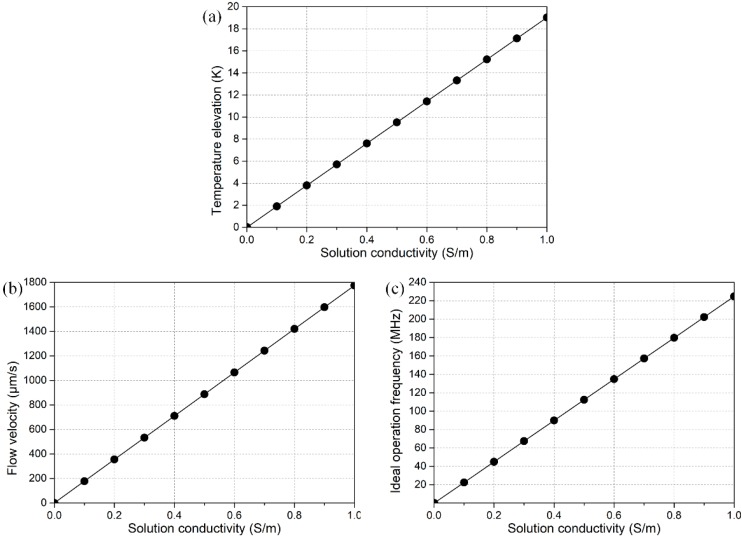
Numerical prediction on conductivity-dependence of the device performance, including (**a**) the maximum temperature elevation; (**b**) electrothermal flow velocity; and (**c**) ideal operation frequency.

As shown in Figure 7, raising the electrical conductivity σ of buffer solution would increase the internal heating ($\Delta T\propto \sigma E^{2}$, Figure 7a), and thereby result in acceleration of electrothermal fluid motion in rotating electric fields ($u\propto \sigma E^{4}$, Figure 7b), while with a compromised trade-off in a sharp increase of the ideal operation frequency *f* = *σ*/2*πε* of the microfluidic device (Figure 7c), which poses a higher demand for the effective working frequency range of the multiphase function generator.

## 4. Conclusions

In summary, we have experimentally investigated the behavior of ROT-ETF in this work, as produced from a four-phase circulating electrode array on a glass substrate. It is discovered that the central out-of-phase TWET whirlpool above the cross-shaped interelectrode gaps rotates in the direction of signal-phase propagation, which violates the actuation of anti-field induction EHD on a glass base predicted by previous researchers [42]. Different heat transfer models are thereby developed to find out the appropriate reasons behind this peculiar flow reversal phenomenon. In fact, from a physical point of view, the importance of electrode cooling in ROT-ETF due to external natural convection has to be addressed when metal electrodes of high thermal conductivity and substrate material of low heat dissipation capability are employed. With an improved heat transfer model incorporating external natural convection on the millimeter-scale electrode pad patterns, the Joule heating-induced hot spots shift from the electrode plane to a bit of vertical distance above the channel bottom surface, resulting in the rectified transition of out-of-phase induction vortex from repulsion-type in the absence of cooling heat flux to attraction-type with electrode cooling effect. Moreover, flow velocity of the horizontal TWET whirlpool predicted under the cooling condition further approaches the experimental measurements because of a lower temperature elevation and hence reduced structural polarization of the internal bulk medium. Our results indicate that the exchange heat flux between ambient atmosphere and conductive electrodes is of great importance in adjusting the direction, pattern as well as velocity of induction EHD flow, providing useful guidelines for electrokinetic manipulation of micro/nano-particle samples using rotating electric fields in microfluidics.

## Figures and Tables

**Table 1:** Nomenclature of mathematical symbols invoked in this study.

Symbol	Implication	Value or Unit
ϕ	Electrostatic potential field	[V]
***E***	Electric field vector	[V/m]
*T*	Temperature field	[K]
*p*	Hydrostatic pressure field	[Pa]
***u***	Flow velocity vector	[m/s]
*ρ_f_*	Volumetric free charge distribution	[C/m^3^]
*ρ_b_*	Volumetric bound charge distribution	[C/m^3^]
*f_ET_*	Electrothermal body force	[N/m^3^]
Δ*T*	Temperature elevation within the bulk fluid	[K]
ε	Liquid permittivity	7.08 × 10^−10^ [F/m]
σ	Electrolyte conductivity	0.05 [S/m]
*f*_c1_	Charge relaxation frequency	σ/2πε = 11 [MHz]
*τ*	Charge relaxation time	ε/σ = 1.42 × 10^−8^ [s]
*f*_c2_	Characteristic crossover frequency of SWET	$\sqrt{5}\sigma /2\pi \varepsilon$ = 24.6 [MHz]
*f*	Field frequency	1~35 [MHz]
*ω*	Angular field frequency	2π*f*
*d*	Nearest distance between oppositely polarized electrodes	50 [μm]
*A*	AC voltage amplitude	[V]
*E_0_*	Electric field magnitude	2*A*/*d*
T_0_	Temperature of reference	293.15 [K]
k_water_	Thermal conductivity of water	0.6 [W·m^−1^·K^−1^]
k_PDMS_	Thermal conductivity of PDMS lid	0.2 [W·m^−1^·K^−1^]
k_glass_	Thermal conductivity of glass substrate	1 [W·m^−1^·K^−1^]
k_Si_	Thermal conductivity of silicon material	140 [W·m^−1^·K^−1^]
k_Au_	Thermal conductivity of gold electrodes	340 [W·m^−1^·K^−1^]
η	Dynamic viscosity of water	0.001 [Pa·s]
H	Thickness of the fluid layer	500 [μm]
H_Sub_	Thickness of glass substrate	500 [μm]
H_Lid_	Thickness of PDMS lid	100 [μm]

## Supplementary Materials

The following are available online at www.mdpi.com/2072-666X/8/11/327/s1.
